# A Systematic Review on Machine Learning Techniques for Survival Analysis in Cancer

**DOI:** 10.1002/cam4.71375

**Published:** 2025-11-20

**Authors:** Autumn O'Donnell, Michael Cronin, Shirin Moghaddam, Eric Wolsztynski

**Affiliations:** ^1^ School of Mathematical and Statistical Sciences University of Galway Galway Ireland; ^2^ Insight SFI Centre for Data Analytics Dublin Ireland; ^3^ School of Mathematical Sciences University College Cork Cork Ireland; ^4^ Department of Mathematics and Statistics (MACSI) University of Limerick Limerick Ireland; ^5^ Limerick Digital Cancer Research Centre (LDCRC) University of Limerick Limerick Ireland; ^6^ Health Research Institute (HRI), University of Limerick Limerick Ireland

**Keywords:** cancer, machine learning, survival analysis, systematic review

## Abstract

**Background:**

Machine learning (ML) offers opportunities to overcome limitations of conventional survival analyses, which are commonly found in cancer studies. However, the choice and levels of performance of ML methods vary across studies. As a result, it is unclear whether they consistently outperform traditional statistical methods and whether one particular ML strategy may outperform others for survival analysis. The present study aimed to systematically review the literature around this emerging topic.

**Methods:**

This systematic review was conducted using the PRISMA guidelines. Electronic databases were systematically searched using keywords related to Machine Learning, Survival Analysis and Cancer.

**Results & Conclusion:**

The review included 196 studies, from which 39 comparable studies were used for the analysis. Improved predictive performance was seen from the use of ML in almost all cancer types. Predictive performance of the different ML methods varied across cancer types, and multi‐task and deep learning methods appeared to yield superior performance; however, it was reported in only a minority of papers. This study also highlighted great variability in both methodologies and their implementations.

**Abbreviations**
CPH: Cox proportional hazard
ML: machine learning

## 1 Introduction

Cancer is one of the most common diseases worldwide, according to the World Health Organization (WHO), with one in five people being diagnosed with cancer in their lifetime and more than 19 million new cases of cancer diagnosed each year. One in 10 people dies from cancer, with approximately 10 million cancer‐related deaths each year [1]. Various factors such as genetic predisposition, environmental influences, lifestyle choices and infections contribute to the development and progression of cancer [2]. Many cancers are preventable and treatable if detected early. Diagnostic tools like the Papanicolaou test (pap smear) or human papillomavirus (HPV) detection test for cervical cancer [3], mammograms for breast cancer [4] and digital rectal examinations (DRE) for prostate cancer [5] are key for early detection. Other tools such as nomograms can be used to help determine the risk of death or cancer progression by using various factors and help to determine suitable treatment pathways [6]. For this reason, much research has been undertaken to determine accurate survival prediction based on various patient information. From a clinical standpoint, this evaluation could be based on a clinician's medical expertise alone or using a clinical nomogram that combines diagnostic or prognostic tools to aid in the decision‐making process [7]. Such models are developed using fine‐tuned mathematical and statistical techniques. As such, survival analysis is a predominant methodological tool in cancer research. Traditional statistical methodologies for survival analysis have limitations, such as linearity assumptions and issues pertaining to high dimensionality, which machine learning (ML) methods have been developed for and adapted to overcome towards improved prediction. There is now a large array of ML methodologies for survival analysis implemented in cancer research, and while their performance varies, it is not clear whether any, or which ML techniques may yield superior performance, or whether they are all capable of improving on traditional methods. This motivated this systematic review, which aimed to evaluate published studies on machine learning models for survival analysis in cancer.

Several reviews and surveys on machine learning for survival analysis exist, but these are not systematic in design and are not specific to cancer, limiting their scope [8, 9]. A systematic review by Deepa and Gunavathi (2022) addressed cancer survival prediction but focused primarily on classification rather than the survival analysis of longitudinal data [10]. Our study is, to our knowledge, the first systematic review dedicated to machine learning and deep learning methods for cancer survival analysis. By applying rigorous selection criteria and focusing specifically on time‐to‐event outcomes, this review provides insight into the comparative performance of machine learning, deep learning and classical statistical approaches.

## 2 Background

### 2.1 Survival Analysis

Survival analysis involves statistical methods to analyse time‐to‐event data where the outcome variable is a clinical endpoint, such as the time (*T*) to death or to recurrence of the disease, with probability density function *f* (*t*) [11]. One of the main functions of interest in survival analysis is the survival function, which models the probability that a subject survives beyond time *t* > 0,

$$S\left(T\right)=\mathrm{Pr}\left(T>t\right)$$

It is a non‐increasing function with *S*(0) = 1 and *S*(*∞*) = 0. Rather than directly estimating the survival function, the hazard function

$$h\left(t\right)=\lim_{∆t\to 0}\frac{p\left(t\leq T<t+∆t|T\geq t\right)}{∆t}=\frac{f\left(t\right)}{S\left(t\right)}$$

is often considered instead, which represents the instantaneous rate at which events occur, given no prior event, with corresponding cumulative hazard function

$$H\left(t\right)=\int_{0}^{t}h\left(u\right)\mathrm{du}$$

such that *S*(*t*) = exp(–*H*(*t*)).

Direct estimation of the survival function may be carried out using a parametric model, assuming a specific distribution for survival times (e.g., exponential, Weibull). Such techniques can be restrictive and may not fit the data well [12]. Time‐to‐event observations are usually right‐censored, as events of interest (e.g., death) may not occur by the end of the study period. This makes direct estimation of the survival function more complex, as the exact survival times for censored observations are unknown. Non‐parametric estimators such as the Kaplan–Meier and Nelson–Aalen methods [13] were developed to handle right‐censored data, providing estimates of survival or cumulative hazard functions under this censoring. It is also the case for other estimators of *S*(*t*), *H*(*t*) or functions thereof, such as Breslow's estimator [14, 15], which are commonly used within semi‐parametric models of the impact of risk variables on survival characteristics of interest.

A common methodology used to assess the role of biomarkers or risk variables in survival that takes censoring into account is the Cox proportional hazards model (CPH) [16]. Assuming a dataset {(*Y*_1_, *X*_1_, *δ*_1_), …, (*Y*_*n*_, *X*_*n*_, *δ*_*n*_)} where *Y*_*i*_, the observed time, is a time‐to‐event if *δ*_*i*_ = 1 or a censored time if *δ*_*i*_ = 0 and *X*_*i*_ is a vector of covariates (*X*_*i*1_, *X*_*i*2_, …, *X*_*ip*_), let *t*_1_ < *t*_2_ < ⋯ < *t*_*m*_ be the increasing list of unique event times, and let *j*(*i*) denote the index of the observation incurring an event at time *t*_*i*_. The CPH model assumes a semi‐parametric form for the hazard

$$h_{i}\left(t\right)=h_{0}\left(t\right)e^{x_{i}^{T}\beta }$$

where *h*_*i*_(*t*) is the hazard for subject *i* at time *t*, *h*_0_(*t*) is a shared baseline hazard and *β* is a fixed *p*‐valued vector with inference made by partial likelihood

$$L\left(\beta \right)=\prod_{i=1}^{m}\frac{e^{x_{j\left(i\right)}^{T}}\beta }{\sum_{j\in R_{i}}e^{x_{j}^{T}\beta }}$$

where *R*_*i*_ is the set of indices *j* with *y*_*j*_ *≥ t*_*i*_ (those at risk at time *t*_*i*_). This semi‐parametric approach does not require a parameterised characterisation of the hazard function, which makes it more robust and widely applicable compared to parametric models of *S*(*t*). However, the assumption of the proportional hazards that it relies upon implies that the hazard ratios between different levels of covariates are constant over time, that is, the effect of covariates on the hazard rate is multiplicative and does not change. The model may provide biased or misleading results if this assumption is violated. The CPH model also relies on the assumption of a log‐linear relationship between the covariates and the hazard function. The model may not fit the data well if the true relationship between covariates and the hazard is non‐linear. While this can be partially addressed by transforming covariates or including interaction terms, it still requires careful consideration and validation [17]. The addition of time‐varying covariates or stratification can alleviate proportional hazards assumption [18]; however, it remains restricted to low‐dimensional data. Cancer research often involves high‐dimensional data, such as electronic health records (EHR), genomic or other molecular data. These datasets typically have many more variables than observed events (censored or uncensored survival times), which renders the CPH model unidentifiable, yielding unstable hazard ratio estimates [19]. Even in situations with a lower, yet still large number of covariates relative to the number of events, the CPH model may overfit the data, leading to unstable estimates [20].

### 2.2 Machine Learning for Survival Analysis

Machine learning methods are well suited for analysing high‐dimensional data often encountered in survival analysis, such as genomic data [21], radiology data [22] or electronic health records (EHR) [23]. ML techniques also have the potential to overcome many of the limitations that traditional statistical methods impose [24] by capturing complex, non‐linear relationships between covariates and survival outcomes, unlike traditional statistical models [25]. Several strategies have already been adapted for survival analysis and were found throughout this systematic review. These include regularisation of the CPH model, approaches based on survival trees, boosting algorithms, support vector machines, shallow neural networks and deep learning.

#### 2.2.1 Regularisation Methods

Regularised alternatives to the conventional CPH model have been developed to allow for the use of the model in high‐dimensionality settings by adding a penalty term to the likelihood function. The least absolute shrinkage and selection operator (LASSO) adds an *L*_1_ penalty to the partial likelihood function, that is, penalises the sum of the absolute values of the coefficients in [1], to an extent that is controlled by a smoothing parameter *λ* > 0 to yield the additive term

$$\lambda \sum_{j=1}^{p}\left|\beta_{j}\right|$$

in the objective function. This approach encourages sparsity in the model [26] by automatically selecting important covariates and shrinking other coefficients toward zero, effectively reducing model complexity. Ridge regression is another regularised approach that instead adds an *L*_2_ penalty to the objective function, that is, penalising the sum of the squared values of the coefficients with the additive term

$$\lambda \sum_{j=1}^{p}\beta_{j}^{2}$$

in the objective function. Although this approach also penalises large coefficients [27], it does not allow shrinkage to make any of the coefficients equal to zero, unlike the LASSO. Elastic net (EN) combines *L*_1_ and *L*_2_ penalties linearly into the penalty term

$$\lambda \sum_{j=1}^{p}|\beta_{j}|+\left(1-\lambda \right)\lambda \sum_{j=1}^{p}\beta_{j}^{2},\lambda \in \left(0,1\right),$$

thus allowing both variable selection and coefficient shrinkage [27]. In any case, the regularisation hyperparameter *λ* thus controls the strength of the penalty applied to the conventional CPH criterion.

#### 2.2.2 Tree‐Based Methods

In tree‐based approaches, the survival outcome is predicted by recursively partitioning the data into subgroups of more comparable risks [28]. At each split, the covariate that maximises a chosen separation criterion is selected to make the split, and the same covariate may be considered for more than one split. Commonly implemented criteria to determine child nodes include:

- maximising the log‐rank test statistic to yield the greatest difference between survival curve estimates of the two subsets [29];
- maximising the likelihood ratio test statistic for exponential survival times [30];
- maximising the Cox partial likelihood [30].

Ensembles of survival trees by random survival forests (RSF) [31] are more commonly used than a single‐tree design. This ensemble relies on bootstrap aggregation, whereby a subset of features is randomly selected at each node to perform the next split. The predicted survival time (or hazard) for each observation is obtained by aggregating predictions from all trees in the forest, which mitigates the risk of overfitting of a single‐tree model [32], and also reduces prediction variability.

An alternative tree‐based method uses conditional inference tests to determine the best split [33]. Thus, the output of the resulting conditional inference tree is the set of conditional survival probabilities determined by the terminal nodes in which each observation is classified. A conditional survival forest (CSF) is an ensemble of conditional trees.

#### 2.2.3 Boosting Methods

Boosting is an ensemble technique that combines the predictions from multiple base learners to improve overall predictive performance. Gradient boosting is a widely used boosting technique that sequentially builds an ensemble of weak learners. Two main methodologies are implemented for boosting in survival analysis: model‐based boosting [34] and likelihood‐based boosting [35]. In model‐based boosting, after an initial model is derived, the residuals are computed, and a weak learner is fit to the residual. This new learner is scaled by the learning rate parameter and linearly combined to the initial model. Each subsequent learner is fit to the errors (residuals) of the previous model, and the process is repeated for a predefined number of iterations or until convergence. In likelihood‐based boosting, the weak learners are fitted by the negative gradient of the log‐likelihood with respect to the parameters of the current model.

Boosting in survival analysis can follow the traditional tree‐based structure similar to the original boosting methods, or it can be based on another modelling structure, such as the CPH model. Hyperparameters for such models include the learning rate parameter (*ν*) and either the number of iterations or the stopping criterion. With an increasing number of boosting iterations, performance can be improved but may also lead to overfitting if not combined with regularisation. An early stopping criterion can be included to stop training early if performance on a validation set does not improve for a set number of iterations. A tolerance for the minimum improvement required to continue training can also be implemented to mitigate these common issues.

#### 2.2.4 Support Vector Machines

Support vector machines (SVM) for classification involve the creation of hyperplane decision boundaries to separate data into classes. In support vector regression (SVR), the implemented hyperplane is the regression function (e.g., a regression line) [36]. The data points on either side of the hyperplane closest to the hyperplane are called support vectors and define the boundary line. SVR consists of fitting the best boundary within an acceptable distance of the hyperplane.

There are two main implementations of survival SVM (SSVM): the regression approach and the ranking approach [37]. The former considers censoring when formulating the inequality constraints of the support vector problem. A linear function that assumes a linear relationship between the covariates and the survival time can be used in the regression approach. Kernel functions can be used to capture non‐linear relationships between covariates and survival time. SVMs use kernel functions to map input features into a higher dimensional space where a hyperplane separates the data non‐linearly. Common kernels include linear, polynomial, radial basis function (RBF) and sigmoid. In the ranking approach, the inequality constraints set the objective to maximise the concordance index for comparable pairs of observations. The objective is to minimise ranking loss, ensuring that the predicted risk scores respect the observed survival order. A hybrid approach can also be implemented, combining the regression and ranking constraints in the same model. The hyperparameters for SSVM include the regularisation parameter, kernel type and kernel parameters.

#### 2.2.5 Neural Networks

Neural networks (NN) for survival analysis leverage the potential of artificial neural network architectures to predict time‐to‐event outcomes and model complex, non‐linear relationships in data. An NN is composed of interconnected nodes (neurons) arranged into an input layer, a number of hidden layers and an output layer. The input layer can take covariates of any type; some NN architectures (convolutional neural networks, CNN) allow raw image data to be taken as input. Each connection between neurons has an associated weight, and each neuron applies an activation function to its input to produce an output ‘proportional hazards’ layer, comparable to the CPH model. The activation function can take one of several forms, the ReLU, sigmoid or tanh functions being common choices, to introduce non‐linearity into the model. Cox‐nnet is an artificial neural network (ANN) with a single hidden layer, its output layer consisting of a CPH [38].

Deep learning is a subset of machine learning focused on neural networks with a large number of hidden layers (deep architectures) and are capable of learning hierarchical features from data. A popular deep feed‐forward neural network, DeepSurv [39], is similar in design to the previously described Cox‐nnet. DeepSurv combines CPH regression with neural networks, training the model so as to optimise the average negative log partial likelihood [39]. In this setting, the final layer is a single node, and the output of the network is taken as the predicted log‐risk function. DeepSurv, like many deep learning methods, also has the potential to be implemented in shallow frameworks. An alternative methodology involves direct modelling of the discrete‐time hazard function, such as the DeepHit model [40]. The output of DeepHit provides the probabilities of different events occurring at specific discrete time‐points. Typical hyperparameters in NNs are the number of hidden layers, the number of neurons in each layer and the activation function used.

## 3 Methods

### 3.1 Study Registration

The present study was registered with the International Prospective Register of Systematic Reviews (the full protocol can be found on PROSPERO; Protocol ID: CRD42023391624) and was conducted in accordance with the Preferred Reporting Items for Systematic Reviews and Meta‐Analysis (PRISMA) guidelines [41].

### 3.2 Search Strategy

Electronic databases were searched from inception to 24 February 2023, including PubMed, Scopus, Web of Science, EMBASE and ACM. The searching methodology included terms and synonyms relating to machine learning techniques for survival analysis in cancer:

(Neoplasm OR Cancer OR Carcinoma) AND (Survival Analysis OR time to event) AND (Machine Learning OR Deep Learning)

Studies of patients with cancer or being assessed for risk of cancer development were eligible for this review. Studies were eligible if they evaluated machine learning methodologies and reported an estimate of either the survival function *S*(*t*) = 1 − *P*(*t*), or the hazard function $h\left(t\right)=\frac{f\left(t\right)}{S\left(t\right)}$. Papers were excluded if the studies only reported on classification models. Comparisons to traditional statistical methodologies for survival analysis (e.g., Cox proportional hazard regression, parametric techniques, etc.) were analysed if present. Studies were included when their primary outcome assessed how machine learning methodologies for survival analysis in cancer compared to traditional statistical methods, with respect to predictive performance, stability and repeatability, where applicable. For predictive performance, acceptable metrics included the concordance index, area under the curve (from a receiver operating characteristic curve), decision curve analysis, etc. For stability, acceptable metrics included the mean or median number of variables included across bootstrapping or cross‐validation, the inclusion of a variable in more than a certain cut‐off threshold of bootstraps of cross‐validation sets, etc. For repeatability, acceptable frameworks included internal or external validation. Information was synthesised where possible. Studies were included when their outcome assessed which machine learning methodology had the greatest predictive performance for survival analysis in cancer, with respect to predictive performance. The following study designs were included: randomised controlled trials, observational studies (all types), cohort (longitudinal) studies, cohort studies, case–control studies, before‐after studies or cross‐sectional studies.

### 3.3 Data Extraction

We created a data extraction form for study characteristics and outcome data piloted by three investigators on 10 randomly selected studies in the review. Data extraction was conducted by AOD. The following data for study characteristics and outcomes were extracted from each included study: Study authors, year, title, study type, data type, data accessibility, software, replicability, pre‐filtering methods applied, additional feature selection methods, sampling framework, cancer/carcinoma type, patient inclusion/exclusion criteria, sample size, interventions and comparators, survival outcome(s) of interest, ML method(s) implemented for survival analysis, reason(s) for the use of ML, type of comparator, traditional method(s) implemented for survival analysis, quantitative comparison of machine learning methodologies for survival analysis in cancer against conventional statistical methods with respect to predictive performance, stability and repeatability, where applicable. Information was synthesised where possible.

### 3.4 Quality Assessment

Due to the nature of this review, the risk of bias was assessed using the ChAMAI checklist [42]. Being focused on a methodological review, the risk of bias was assessed based on the validity of the statistical approach implemented in a paper, the framework it was built upon and the results garnered.

## 4 Results

**FIGURE 1:**
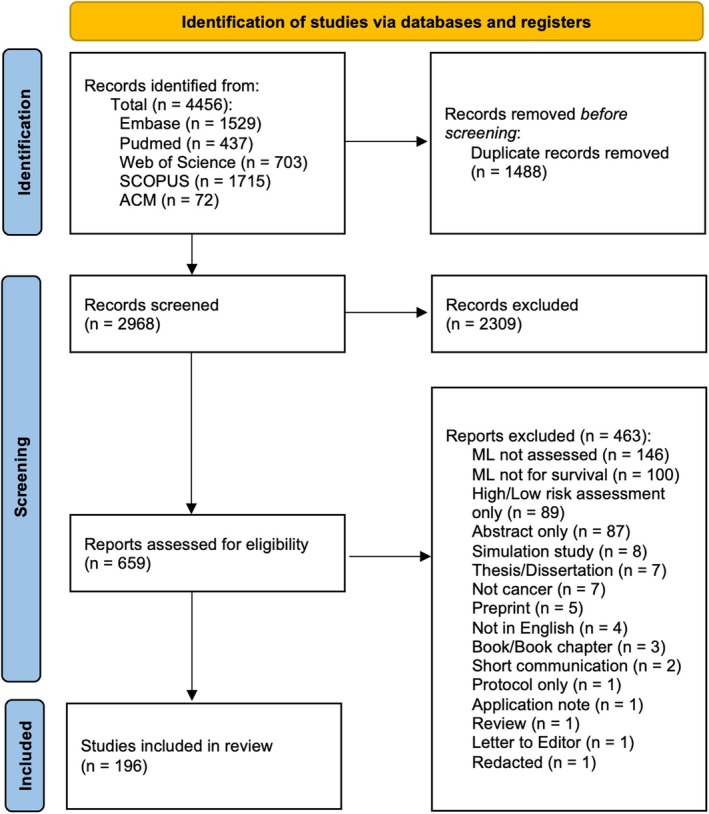
PRISMA flowchart of included studies.

**FIGURE 2:**
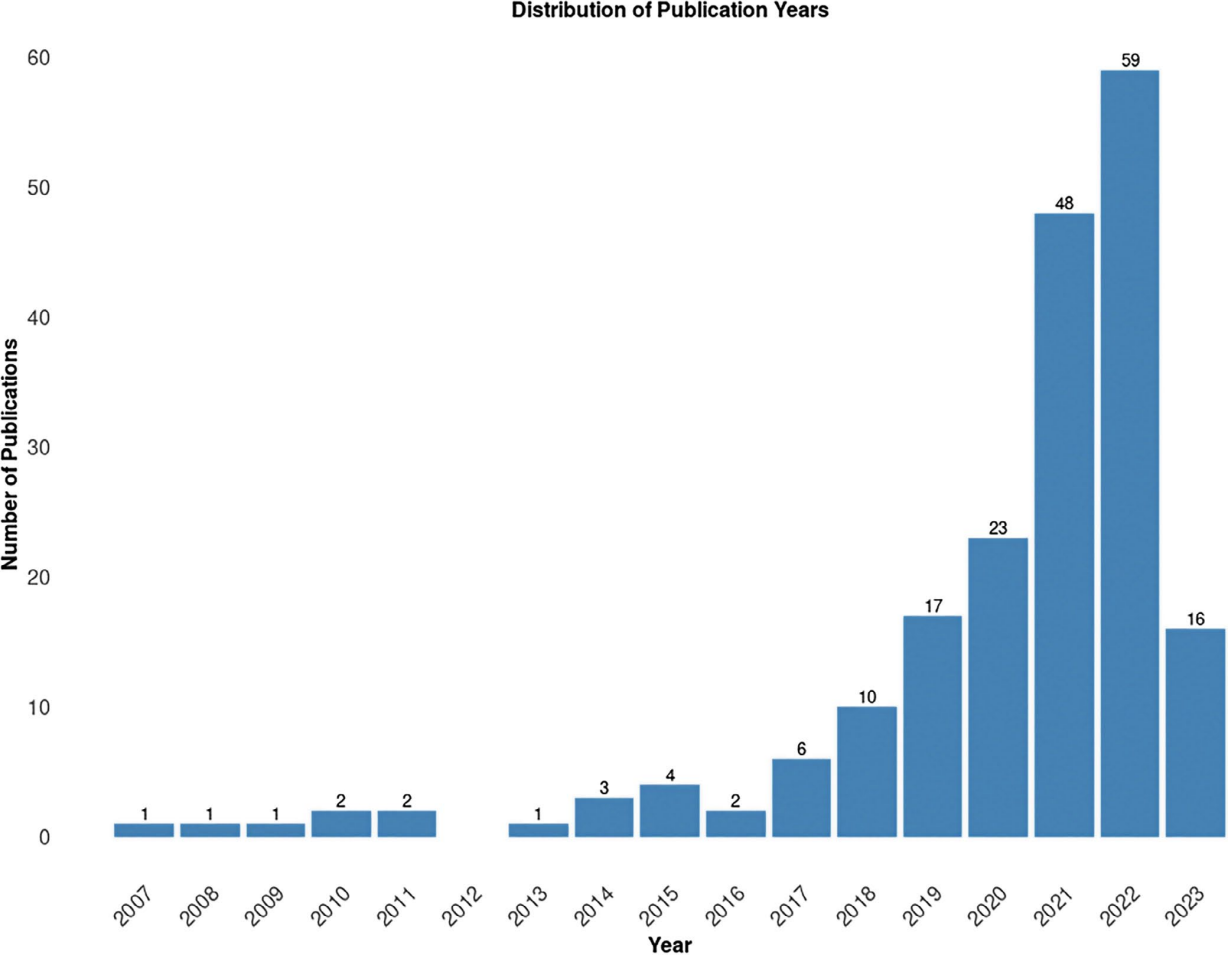
Number of papers by publication year.

In total, 4456 studies were identified from the database searches. After the removal of 1488 duplicates, and another 2309 studies excluded based on their title and abstract, a total of 659 studies were selected for full‐text review. Of these, 463 were excluded (full exclusion reasons are broken down and can be seen in the PRISMA flow diagram, Figure 1). A total of 196 studies were thus included following full‐text review. Table 1 provides a general view of the included studies. Due to inherent differences in cancer types, we have reported on each cancer type individually as appropriate. Thirty‐nine of the included studies considered more than one cancer type in their analysis, five of which considered pan‐cancer. The most prevalent single cancer type studied was lung cancer, accounting for 15% of studies and was also included in 23 studies that considered multiple cancer types. All studies were published between 2007 and 2023, with approximately 75% of the publications from 2020 onwards (Figure 2).

**TABLE 1:** Extracted characteristics of the included studies.

Cancer type	Count		Retrospective		Accessible data		Available source code		Stated time‐point of evaluation		Outcome of interest					
											Multiple outcomes		Overall survival		Other	
	*N*	%	*N*	%	*N*	%	*N*	%	*N*	%	*N*	%	*N*	%	*N*	%
Overall	196	100.0%	188	95.9%	139	70.9%	59	30.1%	56	28.6%	38	19.4%	146	74.5%	81	41.3%
Bladder	6	3.1%	6	100.0%	6	100.0%	2	33.3%	1	16.7%	4	66.7%	3	50.0%	4	66.7%
Blood	8	4.1%	8	100.0%	5	62.5%	2	25.0%	1	12.5%	2	25.0%	8	100.0%	2	25.0%
Bone	2	1.0%	2	100.0%	1	50.0%	1	50.0%	1	50.0%	0	0.0%	2	100.0%	0	0.0%
Brain	16	8.2%	15	93.8%	8	50.0%	2	12.5%	7	43.8%	4	25.0%	15	93.8%	5	31.3%
Breast	22	11.2%	22	100.0%	15	68.2%	11	50.0%	4	18.2%	3	13.6%	15	68.2%	10	45.5%
Cervical	5	2.6%	4	80.0%	4	80.0%	0	0.0%	2	40.0%	1	20.0%	4	80.0%	2	40.0%
Colorectal	11	5.6%	11	100.0%	8	72.7%	0	0.0%	5	45.5%	2	18.2%	4	36.4%	8	72.7%
Oesophageal	2	1.0%	2	100.0%	1	50.0%	0	0.0%	0	0.0%	1	50.0%	1	50.0%	2	100.0%
Gastric	8	4.1%	8	100.0%	4	50.0%	3	37.5%	2	25.0%	1	12.5%	7	87.5%	2	25.0%
Head & Neck	20	10.2%	17	85.0%	13	65.0%	2	10.0%	8	40.0%	6	30.0%	9	45.0%	16	80.0%
Kidney	4	2.0%	4	100.0%	3	75.0%	0	0.0%	1	25.0%	1	25.0%	3	75.0%	1	25.0%
Liver	6	3.1%	6	100.0%	2	33.3%	0	0.0%	4	66.7%	0	0.0%	4	66.7%	2	33.3%
Lung	23	11.7%	21	91.3%	19	82.6%	6	26.1%	10	43.5%	3	13.0%	16	69.6%	10	43.5%
Melanoma	6	3.1%	6	100.0%	5	83.3%	4	66.7%	1	16.7%	1	16.7%	5	83.3%	2	33.3%
Ovarian	2	1.0%	2	100.0%	1	50.0%	1	50.0%	0	0.0%	0	0.0%	1	50.0%	1	50.0%
Pancreatic	3	1.5%	3	100.0%	1	33.3%	0	0.0%	1	33.3%	1	33.3%	3	100.0%	1	33.3%
Prostate	8	4.1%	7	87.5%	6	75.0%	4	50.0%	4	50.0%	2	25.0%	3	37.5%	6	75.0%
Sarcoma	2	1.0%	2	100.0%	0	0.0%	1	50.0%	2	100.0%	1	50.0%	2	100.0%	1	50.0%
Uterine	2	1.0%	2	100.0%	1	50.0%	0	0.0%	1	50.0%	0	0.0%	2	100.0%	0	0.0%
Other	1	0.5%	1	100.0%	0	0.0%	0	0.0%	1	100.0%	1	100.0%	1	100.0%	1	100.0%
MCT	39	19.9%	39	100.0%	36	92.3%	20	51.3%	0	0.0%	4	10.3%	38	97.4%	5	12.8%

Abbreviation: MCT, multiple cancer type.

### 4.1 Data

Across all cancer types, most of the studies were retrospective (188), but eight studies were prospective. The sample size used for analysis varied considerably across the studies, ranging from 17 to over 250,000.

#### 4.1.1 Data Availability

Data availability is vital for advancing the research field and thus improving clinical practice. As previously discussed, with an increase in the dimensionality of data, it becomes more challenging to produce models without an increase in the sample size. By making data available, more accurate models can be created. Approximately 70% of the studies used publicly available data or would be made available by the authors upon request. Twenty percent of studies did not make a statement regarding data availability. Another 3% of studies stated that the data would not be available.

#### 4.1.2 Data Sources

Approximately 50% of the studies used datasets from at least one of the following major public databases: the Cancer Genome Atlas (TCGA), National Center for Biotechnology Informations Gene Expression Omnibus (NCBI GEO), the National Cancer Institutes Surveillance, Epidemiology and End Results (SEER) database, The Cancer Imaging Archive (TCIA) and The National Cancer Database (NCDB). Thirty percent of the studies used hospital‐level data, 7% used other national or global databases as their sole data source, and 3% used clinical trial data. Ten percent of the studies used datasets available in R and Python from packages such as survival, Bioconductor, ipred, DeepSurv, penalised, rotsf, biospear and RankDeepSurv. Eight studies did not state where their data was acquired from.

#### 4.1.3 Data Types

A variety of data types were used across the included studies. Fifty percent of the studies included a single data type, while the remaining contained two or more. The most common data type used was clinicopathological data. Clinicopathological variables were the most prevalent data type across the studies, used in 157 studies (68 on their own, and 89 in combination with other data types). Seventy studies included genomic, transcriptomic or proteomic data alone or in combination with each other or other data types. Forty studies included radiomics data, and 23 studies included raw imaging data.

#### 4.1.4 Study Populations

In order to be able to show the benefit of a designed model, it is necessary to understand the study population on which it has been built and, if assessed, the population it has been validated with. Across all cancer types, approximately 50% of studies reported patient‐level inclusion and exclusion criteria, 25% of studies provided detail at a cohort level and the remaining 25% did not report details of the study population.

#### 4.1.5 Evaluation Time‐Points

When developing models for survival analysis in cancer, the use‐case of the produced model should be considered as the variables considered applicable will depend on the planned clinical application of the proposed model. If, for example, the aim of the model is to determine what further treatment, if any, may be required following surgical intervention, then pathological variables extracted during the surgery can be included in the model. However, if the model aimed to determine if the surgery was likely to be successful in irradiating the cancer, then only variables available pre‐operatively should be included. Over 70% of the studies did not state the time‐point of evaluation being considered by their models. Of the remaining 30%, the most common evaluation time‐points were post‐operative and preoperative.

#### 4.1.6 Outcomes of Interest

Approximately 20% of the studies considered multiple outcomes of interest in their analysis. Seventy‐five percent of studies investigated overall survival as an outcome of interest. Forty‐one percent of studies considered other outcomes, with progression‐free and recurrence‐free survival being the most common. Three studies did not state their outcome of interest directly.

#### 4.1.7 Source Code

When both the data and source code are available, accurate replication of results to validate findings becomes possible. Seventy percent of the studies did not state whether the code was available. Of the remaining 30% of studies, the code was publicly available via depositories such as Github for 53 while the remaining five studies stated that they would make the code available upon request.

### 4.2 Modelling Frameworks

To better understand the structure of the analysis undertaken in the studies, it was important to consider the modelling framework used. Table 2 provides an overview of the frameworks used. The initial element of the pipeline is the decision to implement pre‐filtering of the data prior to the model building. Next is the sampling framework under which the modelling will occur, which can be a simple train/test split for more robust methods such as cross‐validation of bootstrapping. Finally, once the model has been built, validation should be considered.

**TABLE 2:** Modelling frameworks of the included studies.

Cancer type	Sampling framework												Validation					
	Pre‐filtering		Train/test split		*k*‐fold CV		Bootstrapping		Other		None		Internal validation		External validation		None	
	*N*	%	*N*	%	*N*	%	*N*	%	*N*	%	*N*	%	*N*	%	*N*	%	*N*	%
Overall	90	46.4%	104	53.1%	53	27.0%	8	4.1%	9	4.6%	22	11.2%	169	86.2%	39	19.9%	11	5.6%
Bladder	3	50.0%	2	33.3%	3	50.0%	0	0.0%	0	0.0%	1	16.7%	5	83.3%	1	16.7%	0	0.0%
Blood	5	62.5%	1	12.5%	2	25.0%	2	25.0%	0	0.0%	3	37.5%	6	75.0%	2	25.0%	0	0.0%
Bone	1	50.0%	1	50.0%	1	50.0%	0	0.0%	0	0.0%	0	0.0%	2	100.0%	1	50.0%	0	0.0%
Brain	9	56.3%	11	68.8%	1	6.3%	0	0.0%	2	12.5%	2	12.5%	14	87.5%	3	18.8%	0	0.0%
Breast	13	59.1%	9	40.9%	7	31.8%	1	4.5%	1	4.5%	4	18.2%	18	81.8%	2	9.1%	4	18.2%
Cervical	3	60.0%	3	60.0%	1	20.0%	0	0.0%	0	0.0%	1	20.0%	4	80.0%	1	20.0%	0	0.0%
Colorectal	6	54.5%	5	45.5%	2	18.2%	1	9.1%	1	9.1%	2	18.2%	8	72.7%	1	9.1%	3	27.3%
Oesophageal	1	50.0%	2	100.0%	0	0.0%	0	0.0%	0	0.0%	0	0.0%	2	100.0%	1	50.0%	0	0.0%
Gastric	3	37.5%	4	50.0%	2	25.0%	0	0.0%	2	25.0%	0	0.0%	6	75.0%	3	37.5%	0	0.0%
Head & Neck	9	45.0%	9	45.0%	7	35.0%	1	5.0%	1	5.0%	2	10.0%	17	85.0%	7	35.0%	1	5.0%
Kidney	1	25.0%	1	25.0%	2	50.0%	0	0.0%	0	0.0%	1	25.0%	3	75.0%	1	25.0%	1	25.0%
Liver	4	66.7%	2	33.3%	2	33.3%	0	0.0%	1	16.7%	1	16.7%	5	83.3%	2	33.3%	0	0.0%
Lung	12	52.2%	17	73.9%	5	21.7%	1	4.3%	0	0.0%	0	0.0%	22	95.7%	6	26.1%	0	0.0%
Melanoma	3	50.0%	2	33.3%	2	33.3%	0	0.0%	0	0.0%	2	33.3%	4	66.7%	3	50.0%	1	16.7%
Ovarian	1	50.0%	0	0.0%	1	50.0%	1	50.0%	0	0.0%	0	0.0%	2	100.0%	0	0.0%	0	0.0%
Pancreatic	1	33.3%	2	66.7%	1	33.3%	0	0.0%	0	0.0%	0	0.0%	2	66.7%	1	33.3%	0	0.0%
Prostate	1	12.5%	4	50.0%	2	25.0%	0	0.0%	0	0.0%	2	25.0%	6	75.0%	2	25.0%	0	0.0%
Sarcoma	0	0.0%	2	100.0%	0	0.0%	0	0.0%	0	0.0%	0	0.0%	2	100.0%	0	0.0%	0	0.0%
Uterine	1	50.0%	2	100.0%	0	0.0%	0	0.0%	0	0.0%	0	0.0%	2	100.0%	0	0.0%	0	0.0%
Other	1	100.0%	0	0.0%	0	0.0%	1	100.0%	0	0.0%	0	0.0%	1	100.0%	0	0.0%	0	0.0%
MCT	12	30.8%	25	64.1%	12	30.8%	0	0.0%	1	2.6%	1	2.6%	38	97.4%	2	5.1%	1	2.6%

Abbreviation: MCT, multiple cancer type.

#### 4.2.1 Pre‐Filtering

As discussed in the introduction, ML is often implemented in cases where *p* > *n* due to its greater ability to handle high dimensionality [21]. However, when the number of predictors greatly exceeds the sample size, that is, when *p* ≫ *n*, some form of pre‐filtering can be beneficial to improve model performance [24]. Forty‐six percent of the studies implemented pre‐filtering into their modelling pipeline. The most common methods used included univariate Cox proportional hazard feature selection and correlation‐based pre‐filtering, implemented by 29 and 18 studies, respectively. Other pre‐filter methods included the use of predefined or hand‐selected feature sets, pathway analysis, variance or expression‐based thresholding and clustering or principal component analysis.

#### 4.2.2 Resampling Framework

Across all cancer types, 53% of the studies used a train/test split as their sampling framework, while 27% used a *k*‐fold cross‐validation (CV) framework. Another eight studies used bootstrapping for their sampling framework. Another nine studies used other sampling methods, such as Monte‐Carlo CV and modified versions of the cross‐validation framework. More than 10% of studies did not implement any resampling framework or even perform a single data split.

#### 4.2.3 Validation

Due to the nature of the resampling frameworks implemented, the majority (85%) of studies included internal validation of their results. Twenty percent of the studies included external validation, half of which also had internal validation. Eleven studies did not provide any validation of their findings, however and only reported on the training outcomes.

### 4.3 Models

#### 4.3.1 Comparison With Traditional Models

Approximately 55% of the studies compared ML models to a traditional statistical methodology or a current state‐of‐the‐art model (SoA). Half of these included a single ML model, and the other half carried out a comparison involving multiple modelling approaches. Across all studies, 25% considered a single ML model framework and so also did not make any comparisons to a traditional or SoA method (thus, these were not analysed in the primary outcome in this review).

#### 4.3.2 Machine Learning Models

**FIGURE 3:**
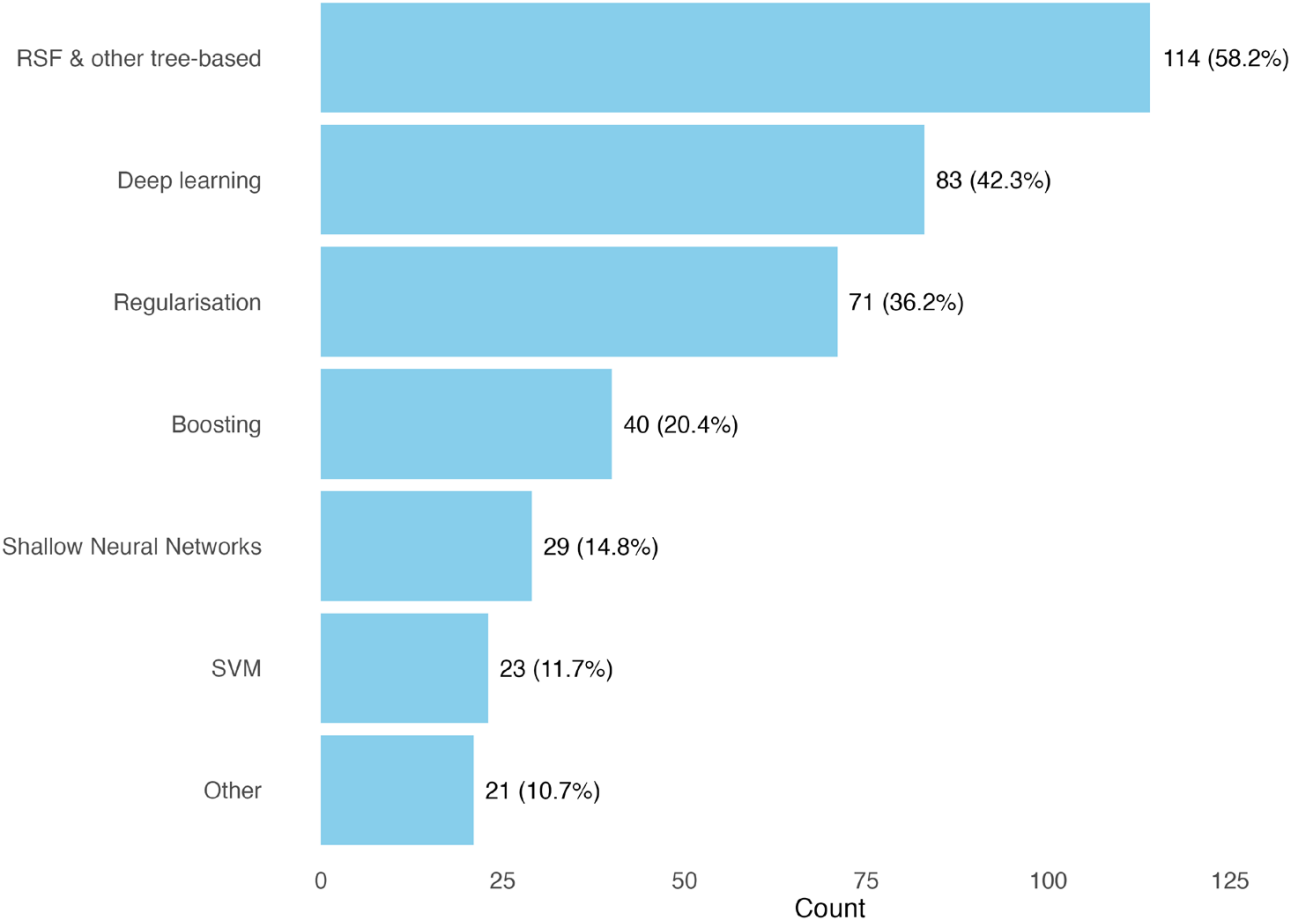
Distribution of machine learning methods implemented across the 196 studies reviewed. Counts indicate the number of studies using each method, and percentages show the proportion of all studies. Note that some studies implemented multiple methods, so percentages may sum to more than 100%.

Figure 3 shows an overview of the ML methods used across the studies, and Table 3 details the breakdown of the implemented modelling strategies. The most prevalent ML modelling method was random survival forest (RSF), which was considered in more than 55% of the studies and as the only ML model in 19% of studies. Fourteen studies also considered other tree‐based methods, such as single survival trees and conditional survival forests (CSF). Neural network‐based methods were the next most prevalent, most of which being deep learning structures (approximately 40% of all the studies), while shallow architectures were considered in 15% of studies. Regularised models were also implemented in more than 35% of the studies. Boosting and SVM structures were considered less frequently (20% and 12% of studies, respectively), and 10% of studies considered other modelling methods, such as Bayesian frameworks and multi‐task models based on a linear model.

**TABLE 3:** Models of the included studies.

Cancer type	Traditional comparison		Multi ML models implemented		Machine learning													
					RSF and other tree‐based methods		Deep learning		Regularisation		Boosting		Shallow neural network		SVM		Other	
	*N*	%	*N*	%	*N*	%	*N*	%	*N*	%	*N*	%	*N*	%	*N*	%	*N*	%
Overall	104	53.1%	110	56.1%	114	58.2%	83	42.3%	71	36.2%	40	20.4%	29	14.8%	23	11.7%	21	10.7%
Bladder	2	33.3%	5	83.3%	2	33.3%	0	0.0%	3	50.0%	4	66.7%	0	0.0%	0	0.0%	2	33.3%
Blood	4	50.0%	3	37.5%	6	75.0%	0	0.0%	3	37.5%	2	25.0%	0	0.0%	0	0.0%	1	12.5%
Bone	2	100.0%	1	50.0%	2	100.0%	1	50.0%	0	0.0%	0	0.0%	1	50.0%	0	0.0%	0	0.0%
Brain	9	56.3%	4	25.0%	10	62.5%	5	31.3%	3	18.8%	0	0.0%	0	0.0%	0	0.0%	0	0.0%
Breast	14	63.6%	17	77.3%	17	77.3%	9	40.9%	6	27.3%	5	22.7%	2	9.1%	4	18.2%	3	13.6%
Cervical	4	80.0%	3	60.0%	4	80.0%	1	20.0%	2	40.0%	1	20.0%	0	0.0%	0	0.0%	1	20.0%
Colorectal	6	54.5%	3	27.3%	8	72.7%	3	27.3%	3	27.3%	0	0.0%	0	0.0%	0	0.0%	0	0.0%
Oesophageal	1	50.0%	0	0.0%	0	0.0%	1	50.0%	1	50.0%	0	0.0%	0	0.0%	0	0.0%	0	0.0%
Gastric	4	50.0%	4	50.0%	3	37.5%	6	75.0%	1	12.5%	1	12.5%	1	12.5%	1	12.5%	0	0.0%
Head & Neck	7	35.0%	11	55.0%	11	55.0%	9	45.0%	9	45.0%	4	20.0%	5	25.0%	1	5.0%	1	5.0%
Kidney	2	50.0%	2	50.0%	2	50.0%	3	75.0%	1	25.0%	1	25.0%	0	0.0%	0	0.0%	0	0.0%
Liver	2	33.3%	4	66.7%	6	100.0%	3	50.0%	3	50.0%	1	16.7%	0	0.0%	2	33.3%	0	0.0%
Lung	10	43.5%	10	43.5%	9	39.1%	11	47.8%	10	43.5%	4	17.4%	3	13.0%	3	13.0%	0	0.0%
Melanoma	4	66.7%	4	66.7%	4	66.7%	0	0.0%	1	16.7%	3	50.0%	3	50.0%	1	16.7%	0	0.0%
Ovarian	0	0.0%	1	50.0%	2	100.0%	1	50.0%	1	50.0%	1	50.0%	0	0.0%	0	0.0%	1	50.0%
Pancreatic	1	33.3%	1	33.3%	2	66.7%	0	0.0%	0	0.0%	1	33.3%	0	0.0%	1	33.3%	0	0.0%
Prostate	8	100.0%	4	50.0%	4	50.0%	6	75.0%	1	12.5%	1	12.5%	0	0.0%	1	12.5%	2	25.0%
Sarcoma	2	100.0%	1	50.0%	1	50.0%	1	50.0%	0	0.0%	0	0.0%	0	0.0%	0	0.0%	0	0.0%
Uterine	2	100.0%	1	50.0%	1	50.0%	1	50.0%	1	50.0%	0	0.0%	0	0.0%	1	50.0%	0	0.0%
Other	0	0.0%	0	0.0%	1	100.0%	0	0.0%	0	0.0%	0	0.0%	0	0.0%	0	0.0%	0	0.0%
MCT	20	51.3%	31	79.5%	19	48.7%	22	56.4%	22	56.4%	11	28.2%	14	35.9%	8	20.5%	10	25.6%

Abbreviation: MCT, multiple cancer type.

### 4.4 Evaluation

Evaluation metrics were considered under three main headings: discrimination, calibration and predictive performance. These three groups are described below, and Table 4 summarises how the most common metrics in each group were used in the literature reviewed by cancer type.

**TABLE 4:** Distribution of metrics used in the 196 studies reviewed, by cancer type.

Cancer type	Discrimination						Calibration		Predictive performance			
	C‐index		Other		None				AUC		Decision curve analysis	
	*N*	%	*N*	%	*N*	%	*N*	%	*N*	%	*N*	%
Overall	163	83.2%	34	17.3%	25	12.8%	49	25.0%	61	31.1%	6	3.1%
Bladder	3	50.0%	2	33.3%	2	33.3%	2	33.3%	4	66.7%	0	0.0%
Blood	6	75.0%	2	25.0%	1	12.5%	2	25.0%	2	25.0%	0	0.0%
Bone	1	50.0%	1	50.0%	1	50.0%	1	50.0%	2	100.0%	0	0.0%
Brain	13	81.3%	2	12.5%	2	12.5%	2	12.5%	5	31.3%	1	6.3%
Breast	18	81.8%	5	22.7%	3	13.6%	5	22.7%	6	27.3%	0	0.0%
Cervical	4	80.0%	1	20.0%	1	20.0%	1	20.0%	1	20.0%	0	0.0%
Colorectal	10	90.9%	1	9.1%	1	9.1%	4	36.4%	4	36.4%	0	0.0%
Oesophageal	2	100.0%	0	0.0%	0	0.0%	1	50.0%	1	50.0%	0	0.0%
Gastric	8	100.0%	0	0.0%	0	0.0%	2	25.0%	4	50.0%	1	12.5%
Head & Neck	17	85.0%	4	20.0%	3	15.0%	6	30.0%	7	35.0%	0	0.0%
Kidney	4	100.0%	0	0.0%	0	0.0%	0	0.0%	1	25.0%	0	0.0%
Liver	6	100.0%	0	0.0%	0	0.0%	0	0.0%	2	33.3%	1	16.7%
Lung	19	82.6%	3	13.0%	3	13.0%	5	21.7%	6	26.1%	1	4.3%
Melanoma	3	50.0%	0	0.0%	3	50.0%	2	33.3%	2	33.3%	0	0.0%
Ovarian	1	50.0%	1	50.0%	0	0.0%	0	0.0%	0	0.0%	0	0.0%
Pancreatic	3	100.0%	0	0.0%	0	0.0%	0	0.0%	1	33.3%	0	0.0%
Prostate	4	50.0%	1	12.5%	3	37.5%	3	37.5%	1	12.5%	2	25.0%
Sarcoma	0	0.0%	0	0.0%	2	100.0%	1	50.0%	2	100.0%	0	0.0%
Uterine	2	100.0%	1	50.0%	0	0.0%	1	50.0%	0	0.0%	0	0.0%
Other	0	0.0%	1	100.0%	0	0.0%	1	100.0%	1	100.0%	0	0.0%
MCT	39	100.0%	9	23.1%	0	0.0%	10	25.6%	9	23.1%	0	0.0%

Abbreviation: MCT, multiple cancer type.

#### 4.4.1 Discrimination

In the context of survival analysis, discrimination metrics refer to measures that assess the ability of a model to distinguish between individuals with different survival times correctly. As we are generally interested in survival across a period of time rather than at one specific time‐point, the discrimination metrics used to evaluate the model must allow assessment of the cohort over time and, therefore, commonly rely on an evaluation of either *S*(*t*) or *h*(*t*) for each subject.

The most commonly implemented metric was Harrell's concordance index (C‐index) [43] defined as the proportion of all feasible pairs of patients whose predicted and observed outcomes are concordance, was used in over 80% of studies to evaluate their models discriminatory ability. Approximately 15% of which also consider other discriminatory metrics such as the integrated Brier Score (IBS) [44] which is the average of the Brier score [45] values for an interval of time [46]. Four other studies used IBS as the sole metric for discrimination, and four more used other novel evaluation methods. The remaining 25 studies did not evaluate their models using a discrimination metric applicable to longitudinal survival data.

#### 4.4.2 Calibration and Predictive Performance

Calibration metrics evaluate how well the predicted survival probabilities agree with the actual observed outcomes. While discrimination metrics are used to assess risk discrimination across survival times, calibration metrics allow to evaluate accuracy of the predicted probabilities. A quarter of the studies included an evaluation of model calibration; most implemented the Brier Score or calibration curves.

Performance metrics that focus on assessing model performance at specific time‐points rather than over the entire duration of follow‐up allow for evaluation of how well a model predicts the probability of an event occurring by a particular clinical time‐point of interest in the patient follow‐up (e.g., at the 3‐year survival horizon) [47]. Pre‐specified time‐points can be determined for each cancer type to consider someone disease‐free and inform subsequent follow‐up. Other forms of model performance can also be considered, such as classification metrics, including the none time‐dependent measures of receiving operator characteristics (ROC) curve analysis to report on the area under the ROC curve (AUC) [11], as a binary metric evaluated only on uncensored observations.

Almost 40% of studies included additional forms of performance evaluation. Most of these used a form of AUC, either in a time‐dependent assessment or as a binary metric evaluated only on uncensored observations. Six studies used decision curve analysis [48] for net benefit evaluation and specific time‐points. The net benefit considered in the DCA is an indication of the increased or decreased number of patients who could see benefit in their treatment if the model was used for evaluation, where a positive net benefit can be viewed as the proportion of patients who would benefit the from use of the model in a clinical decision making setting.

### 4.5 Stability and Feature Selection

Most ML pipelines include pre‐filtering, inbuilt dimensionality reduction, and/or other feature selection techniques. The primary goal of these steps is usually either to enhance model‐building performance or identify relevant biomarkers (or both). The stability of this feature elimination and selection process should be assessed to evaluate (and ensure) future usability, reliability and interpretability of a given model in clinical settings. The resampling framework, which feature selection also depends upon, can be leveraged to assess this aspect of the model by determining selection rates for each of the candidate features [49], or evaluate specific selection performance metrics [50, 51]. Table 5 reports on the breakdown of this information. Less than 50% of the studies considered in this review reported on the stability of their models or provided an assessment of included features.

**TABLE 5:** Distribution of feature selection approaches used in the 196 studies reviewed, by cancer type.

Cancer type	Variable assessment		Feature selection					
			Supervised		Unsupervised		None	
	*N*	%	*N*	%	*N*	%	*N*	%
Overall	87	44.4%	61	31.1%	55	28.1%	104	53.1%
Bladder	5	83.3%	1	16.7%	2	33.3%	3	50.0%
Blood	7	87.5%	5	62.5%	2	25.0%	3	37.5%
Bone	2	100.0%	1	50.0%	1	50.0%	1	50.0%
Brain	8	50.0%	6	37.5%	3	18.8%	7	43.8%
Breast	7	31.8%	7	31.8%	11	50.0%	8	36.4%
Cervical	2	40.0%	3	60.0%	1	20.0%	2	40.0%
Colorectal	7	63.6%	6	54.5%	1	9.1%	5	45.5%
Oesophageal	1	50.0%	1	50.0%	0	0.0%	1	50.0%
Gastric	4	50.0%	2	25.0%	1	12.5%	5	62.5%
Head & Neck	11	55.0%	9	45.0%	5	25.0%	11	55.0%
Kidney	1	25.0%	0	0.0%	1	25.0%	3	75.0%
Liver	3	50.0%	2	33.3%	4	66.7%	2	33.3%
Lung	4	17.4%	10	43.5%	6	26.1%	11	47.8%
Melanoma	1	16.7%	2	33.3%	3	50.0%	3	50.0%
Ovarian	1	50.0%	0	0.0%	1	50.0%	1	50.0%
Pancreatic	2	66.7%	1	33.3%	0	0.0%	2	66.7%
Prostate	3	37.5%	1	12.5%	0	0.0%	7	87.5%
Sarcoma	0	0.0%	0	0.0%	0	0.0%	2	100.0%
Uterine	1	50.0%	1	50.0%	1	50.0%	0	0.0%
Other	1	100.0%	1	100.0%	1	100.0%	0	0.0%
MCT	16	41.0%	2	5.1%	11	28.2%	27	69.2%

Abbreviation: MCT, multiple cancer type.

The nature of the feature selection and/or dimensionality reduction techniques used imposes constraints on their implementation. If the filtering or selection approach is unsupervised (i.e., the outcome variable is not taken into consideration), the filter can be applied to the entire dataset before the implementation of the resampling framework. Considering these methods within a resampling framework or on a separate hold‐out set is also possible, but this is not a requirement. When feature selection/filtering is supervised, however, the outcome variable is used to make feature selection/filtering decisions, and such techniques should be applied only on the training samples (or a separate hold‐out set for preliminary analysis), that is, only on data points not used for model testing or validation, to avoid introducing selection bias into the model [52]. Approximately 50% of the 196 studies reviewed implemented one or more forms of feature selection. Sixty‐one studies used supervised techniques, and 55 used unsupervised methods, 24 of which used both. Note that the number of studies that implement some form of feature selection does not directly relate to those that use pre‐filtering, as some of the supervised techniques include recursive feature elimination methods.

## 5 Discussion

In this systematic review, we have summarised the outcomes of 196 studies relating to the use of machine learning methods for survival analysis in cancer. The primary outcome of interest is how these ML methods compare to traditional statistical methods. An additional outcome of interest being which ML method has the greatest predictive performance. In order to make this evaluation relevant to the broadest community, we inspected each cancer type individually and then summarised their combined findings. Studies that looked at multiple cancer types were included in the analysis of each relevant cancer.

### 5.1 Comparison to Traditional Methods and Predictive Performance

Due to the high variability in study frameworks, we focused the analysis for our primary outcome on a subset of studies that evaluated the C‐index of models used for the prediction of overall survival. This consisted of 57 studies, 17 of which considered multiple cancer types. Four of the multiple cancer studies only reported on pan‐cancer analysis, so results could not be separated by cancer type. Fourteen further studies did not report the numeric values of the results, only including a graphical representation of the discussion; thus, these were excluded from the comparative analysis. The remaining 39 studies were analysed to evaluate performance in predicting the outcome of interest. Table 6 shows the breakdown of comparisons by cancer type.

**TABLE 6:** Description and distribution of outcomes discussed in the 196 articles reviewed, by cancer type.

Cancer type	*N*	Framework	References	C‐index							
				CPH	ML methods						
					RSF	Deep learning	Regularisation	Boosting	Shallow neural network	SVM	Other
Bladder	318	CV	Ning, Zhenyuan (2023) [53]	0.566	0.585		0.586			0.598	0.721
	396	CV	Zhao, Zhangxin (2023) [54]	0.549		0.625	0.605				0.679
Blood	129	Train/test split	Van Belle, Vanya (2011) [37]	0.690						0.690	
	116	CV	Gu, Wangrong (2021) [55]	0.644			0.526				0.644
	92	CV	Gu, Wangrong (2021) [55]	0.488			0.684				0.753
	523	CV	Li, Chunyang (2021) [56]	0.640	0.730		0.730				
Bone	3145	Train/test split	Yan, Lizhao (2022) [24]	0.773	0.803	0.832			0.821		
Brain	502	CV	Ning, Zhenyuan (2023) [53]	0.676	0.728		0.712			0.759	0.784
	502	CV	Zhao, Zhangxin (2023) [54]	0.596		0.685	0.696				0.708
	407	CV	Audureau, Etienne (2018) [57]	0.698	0.701	0.700					0.704
	286	Train/Test split	Jiang, Shuai (2021) [58]	0.774		0.715					
	95	Train/test split	Chen, Haiyan (2021) [59]	0.811	0.847					0.823	
	209	Train/test split	Tang, Bo (2019) [60]	0.506		0.670	0.600	0.598			
	181	Train/test split	Lu, Yiping (2020) [61]	0.630	0.908						
	260	Train/test split	Moradmand, Hajar (2021) [62]	0.674	0.668						
Breast	686	Train/test split	Van Belle, Vanya (2011) [37]	0.670						0.670	
	1980	Train/test split	Katzman, Jared L. (2018) [39]	0.632	0.620	0.654					
	2232	Train/test split	Katzman, Jared L. (2018) [39]	0.659	0.648	0.676					
	115	CV	Gu, Wangrong (2021) [55]	0.396			0.557				0.697
	78	CV	Gu, Wangrong (2021) [55]	0.603			0.606				0.722
	295	CV	Gu, Wangrong (2021) [55]	0.536			0.771				0.756
	686	CV	Sundrani, Sameer (2021) [63]	0.737				0.747			
	583	CV	Huang, Zhi (2019) [64]	0.653	0.629	0.692			0.726		
	456	CV	Fu, Xiaohang (2023) [65]	0.632	0.620	0.748			0.643		
	573	CV	Goli, Shahrbanoo (2016) [66]	0.610						0.610	
	1673	Monte Carlo CV	Bice, Noah (2020) [67]	0.625	0.737	0.749					
	154,401	Train/test split	Chi, Shengqiang (2021) [68]	0.791	0.801	0.809		0.764	0.807		
	322,348	Train/test split	Park, Jung In (2023) [69]	0.828				0.813		0.834	0.804
	1980	Train/test split	Jing, Bingzhong (2018) [70]	0.631	0.619	0.661					
	2232	Train/test split	Jing, Bingzhong (2018) [70]	0.658	0.648	0.688					
Cervical	768	CV	Matsuo, Koji (2018) [71]	0.607	0.600	0.616	0.606	0.606			
	85	Train/test split	Carlini, Gianluca (2022) [72]	0.707	0.517						
Colorectal	888	CV	Sundrani, Sameer (2021) [63]	0.647				0.663			
	11,098	Train/test split	Chi, Shengqiang (2021) [68]	0.771	0.774	0.782		0.753	0.780		
	46	Bootstrapping	Karatza, Eleni (2022) [73]	0.606	0.473		0.699				
	128,061	Train/test split	Tian, Yu (2018) [74]	0.731	0.898						
	49,275	Train/test split	Yu, Haohui (2022) [75]	0.788		0.824					
Gastric	3505	Train/test split	Donghia, Rossella (2023) [76]	0.764	0.773					0.545	
	2935	Train/test split	Huang, Tao (2022) [77]	0.693	0.716	0.731		0.709			
	1061	CV	Hao, Degan (2022) [78]	0.696		0.849					
Kidney	405	CV	Ning, Zhenyuan (2023) [53]	0.594	0.630		0.608			0.632	0.699
	230	CV	Schulz, Stefan (2021) [79]	0.747		0.779					
Liver	333	CV	Ning, Zhenyuan (2023) [53]	0.543	0.595		0.545			0.601	0.719
Lung	139	Train/test split	Van Belle, Vanya (2011) [37]	0.680						0.690	
	167	Train/test split	Van Belle, Vanya (2011) [37]	0.610						0.620	
	403	CV	Ning, Zhenyuan (2023) [53]	0.536	0.557		0.540			0.568	0.708
	438	CV	Ning, Zhenyuan (2023) [53]	0.570	0.579		0.588			0.577	0.695
	86	CV	Gu, Wangrong (2021) [55]	0.432			0.708				0.711
	98	Train/test split	Tang, Bo (2019) [60]	0.517		0.673	0.449	0.524			
	28,927	Train/test split	Chi, Shengqiang (2021) [68]	0.707	0.694	0.728		0.690	0.714		
	109	Train/test split	Yang, Bin (2020) [80]	0.729			0.737				
	165	Train/test split	Hong, Duo (2021) [81]	0.698			0.677				
	1137	Train/test split	Wang, Jianyong (2021) [82]	0.561		0.637			0.563		
	2687	Train/test split	Oh, Seungwon (2023) [83]	0.747					0.747		
	16,608	Train/test split	Yang, Linlin (2022) [84]	0.629	0.635	0.665					
Melanoma	367	CV	Ning, Zhenyuan (2023) [53]	0.493	0.515		0.505			0.538	0.653
Ovarian	295	CV	Ning, Zhenyuan (2023) [53]	0.549	0.571		0.555			0.556	0.682
Pancreatic	12,905	CV	Kim, Hyunsuk (2022) [85]	0.644	0.634					0.623	
Prostate	482	Train/test split	Van Belle, Vanya (2011) [37]	0.770						0.760	
	181,249	Train/test split	Chi, Shengqiang (2021) [68]	0.801	0.802	0.805		0.783	0.806		
Uterine	539	CV	Ning, Zhenyuan (2023) [53]	0.566	0.614		0.603			0.626	0.711
	539	CV	Zhao, Zhangxin (2023) [54]	0.544		0.659	0.597				0.711
	1751	Train/test split	Yang, Bin (2020) [80]	0.729			0.737				
	165	Train/test split	Mysona (2020) [86]	0.730	0.730						
	797	Train/test split	Qu, Wenjie (2021) [87]	0.726					0.774		
Thyroid	499	CV	Ning, Zhenyuan (2023) [53]	0.577	0.655		0.664			0.705	0.862

A major finding of this review was that across all cancer types, improved predictive performance can be seen from machine learning methods, with the exception of cervical cancer. Multi‐task‐based methods were seen to perform well across many cancer types. Deep learning approaches also performed well, though when compared to the multitask method, they only had improved performance in kidney cancer. The most common deep learning model implemented was DeepSurv, although when multiple deep learning models were implemented in a study, it was generally outperformed by the other approaches. Although the regularised models generally outperformed the traditional CPH, they had the lowest performance of the ML methods. Of note, with both deep learning and multitask and other methods, the novel approaches introduced by the studies usually showed the best predictive performance. This may be suggestive of bias in the study design and could be partly due to publication bias. Most of the novel methods have also not been used in other studies and so their performances should be investigated outside of the original study for a better understanding of their capabilities. This may be due to the assumptions of linearity and proportionality that the regularised models must adhere to being too restrictive for the data. LASSO regularisation was the most common technique implemented, though when compared to Elastic Net, it was generally outperformed. This may indicate that the *L*_1_ penalisation is overly stringent and potentially reducing too many important coefficients to zero while the more relaxed *L*_2_ penalty element of the elastic net model counteracts this. RSF, boosting methods and SVM all showed varying predictive capabilities across the cancer types. RSF was the most commonly implemented ML method, with at least one study for each cancer type. It was shown to have the best performance across the blood cancer studies and in pancreatic cancer, but other ML methods outperformed it in the other cancer types. It showed improved performance compared to the traditional CPH in the majority of studies; however, in breast and cervical cancer, the CPH model generally remained superior. SVM showed particularly good performance in brain cancer studies, but it was comparable to the CPH model in the other cancer types. Although different from CPH, the assumptions of SVM can still be restrictive depending on the method used and the kernel function implemented. Boosting methods were implemented in very few of the studies. It showed particularly good performance in breast cancer and generally outperformed the CPH model across all the cancer types it was considered in, though other ML methods outperformed it. The boosting methods implemented included mainly GBM and XGBoost but Boosted trees, CoxBoost and BoostCI were each implemented in a study also. Thus, some of the variability seen in the performance of the boosted models may be due to the variability in the modelling structures used.

#### 5.1.1 Comparison With Benchmarking Studies

Previous benchmarking studies have provided valuable evidence on the performance of machine learning methods for survival analysis [88, 89]. Hermann et al. showed that Cox models with clinical variables often perform comparably to more complex ML approaches using multi‐omic high‐dimensional cancer datasets, while Burk et al. made similar findings in low‐dimensional, non‐cancer‐specific settings. Our findings are consistent with these observations, particularly in the context of regularised, tree‐based and boosting methods, which they assessed. However, unlike these benchmarks, our review also considered other modelling approaches, such as deep learning and multi‐task methods, which were not included in Hermann et al.'s and Burk et al.'s analysis, and which we found to show improvement on classical statistical approaches.

### 5.2 Stability and Modelling Structures

When models are trained using a resampling framework such as repeated train/test split, k‐fold CV or bootstrapping, there is potential that the same variables are not selected every time for model training. Determining the stability of variable selection and model training is therefore necessary to understand which variables are being considered consistently. This can be done by inspecting variable selection rates across resamples, or a formal index of similarity could be considered, such as the Jaccard index [50]. Although more than 80 of the studies used a resampling framework for model training (Table 2) only five evaluated stability of their models. Three investigated selection rates [32, 90, 91]. One used the Jaccard Index [92], and one used interclass correlation to assess stability [93]. In summary, model selection/training stability often appears neglected, despite its importance. Particularly in the case of genomic‐based variables, as was discussed in detail by Venet et al. [94], the significance of a variable in a single model does not necessarily mean it is of clinical importance and should be investigated further.

### 5.3 Bias and Quality Assessment

Some particular issues of bias were noted across the studies concerning statistical modelling related to validation and feature selection. As previously mentioned, 11 studies did not implement any form of internal or external validation. Thus, the results from these studies are from the model building and are not able to give a bearing on the models' ability to perform on unseen data. Another issue found in several studies was the use of supervised feature selection techniques, such as univariate CPH, on the entire dataset prior to splitting or CV. This was seen in nine studies, and a further 13 studies did not clearly report on when the supervised feature selection took place; thus, there is potential that these may also have taken place outside the sampling framework. This method of pre‐filtering will result in inflated validation results as the included variables are already known to be predictive of survival in the testing dataset. Other quality assessment issues relate to the clinical usefulness of the models built in the studies. As per the ChAMAI checklist, the description of the study population and analysis of net benefit are key requirements when reporting on machine learning in medical settings. Over 25% of the studies did not report on the study population demographics or their inclusion/exclusion criteria. Without knowledge of the included subjects, the viability in other populations becomes more challenging to determine, and a potential lack of model predictive ability may be due to an unreported underlying characteristic of the study population. Also, only six studies reported on net benefit or decision curve analysis. These metrics help to assess the clinical usefulness of implementing a model into routine practice, and without them, it is difficult to show the true potential of a novel model. Other important elements for the ChAMAI checklist not regularly addressed across the included studies relate to model deployment. Information regarding model interpretability and explainability for the more complex, black‐box ML methods over simpler, transparent classical models, was seldom addressed, with less than 5% of the studies reporting on variable importance plots or Shapley Additive Examinations Plots (SHAP) [95]. Model explainability is of particular importance when considering for use in a clinical setting. It however remains an open challenge, which may explain why it was seldom discussed in the literature reviewed.

## 6 Conclusion

The use of machine learning methods for survival analysis has been increasing rapidly in recent years. In cancer research, the popularity of these methods is due to their ability to handle high dimensionality and non‐linear effects, which allows the use of larger and more complex data and offers opportunities to improve detection and prediction performance. ML is, therefore, now commonly considered, for example, for tumour detection and for disease characterisation, but less so for survival analysis. This systematic review aimed to determine which machine learning methods were being implemented specifically for survival analysis in cancer studies and, from this, garner insight as to whether machine learning had the ability to outperform traditional models. A second objective of this review was to identify which methods, if any, showed superior performance or potential.

This review yielded two major findings. One was that improved predictive performance was obtained from machine learning methods across all cancer types, with the exception of cervical cancer. The other was in the heterogeneity of ML models used, and variability in the choice of ML pipelines. A majority of papers reported on output for a random survival forest or other tree‐based method (58%, 114/196 studies), deep learning frameworks (42%, 83/196 studies) or regularised Cox models (36%, 71/196 studies).

Studies did not consistently compare the ML methods to conventional survival models or always make comparisons between several machine learning models, limiting the number of studies to draw conclusions from. Direct comparisons between machine learning models were also limited by the large variations in outcomes of interest and performance metrics used and reported on. In the subset of 39 studies which quoted the C‐index for models concerned with overall survival, it was found that machine learning methods could outperform the Cox proportional hazard model in most cancer types. Predictive performance of the various ML methods varied across cancer types with multi‐task and deep learning methods generally appearing to yield superior performance.

## Author Contributions

**Autumn O'Donnell:** conceptualization, methodology, formal analysis, writing – original draft, writing – review and editing, investigation, data curation. **Michael Cronin:** writing – review and editing, funding acquisition, formal analysis, supervision. **Shirin Moghaddam:** supervision, formal analysis, writing – review and editing. **Eric Wolsztynski:** supervision, formal analysis, writing – review and editing.

## Conflicts of Interest

The authors declare no conflicts of interest.

## Supporting information


**Data S1:** This file provides an overview of the 196 studies included in the systematic review.

**Data S2:** This file contains the results of the performance analysis, focusing on comparisons of ML and traditional methods for overall survival prediction.

## Data Availability

The data that support the findings of this study are openly available in survivalML_systematicreview at https://github.com/AutumnLou/survivalML_systematicreview.
